# Discharge β-Blocker Use and Race after Coronary Artery Bypass Grafting

**DOI:** 10.3389/fpubh.2014.00094

**Published:** 2014-07-29

**Authors:** Wesley T. O’Neal, Jimmy T. Efird, Stephen W. Davies, Jason B. O’Neal, William F. Griffin, T. Bruce Ferguson, W. Randolph Chitwood, Alan P. Kypson

**Affiliations:** ^1^Department of Internal Medicine, Wake Forest School of Medicine, Winston-Salem, NC, USA; ^2^East Carolina Heart Institute, Department of Cardiovascular Sciences, Brody School of Medicine, East Carolina University, Greenville, NC, USA; ^3^Center for Health Disparities, Brody School of Medicine, East Carolina University, Greenville, NC, USA; ^4^Department of General Surgery, University of Virginia School of Medicine, Charlottesville, VA, USA; ^5^Department of Anesthesia, Critical Care, and Pain Medicine, Beth Israel Deaconess Medical Center, Harvard Medical School, Boston, MA, USA

**Keywords:** outcomes, CABG, epidemiology, β-blockers, cardiology

## Abstract

**Introduction:** The use of discharge β-blockers after cardiac surgery is associated with a long-term mortality benefit. β-Blockers have been suggested to be less effective in black cardiovascular patients compared with whites. To date, racial differences in the long-term survival of coronary artery bypass grafting (CABG) patients who receive β-blockers at discharge have not been examined.

**Methods:** A retrospective cohort study was conducted on patients undergoing CABG between 2002 and 2011. Long-term survival was compared in patients who were and who were not discharged with β-blockers. Hazard ratios (HR) and 95% confidence intervals (CI) were computed using a Cox regression model. P-for-interaction between race and discharge β-blocker use was computed using a likelihood ratio test.

**Results:** A total of 853 (88%) black (*n* = 970) and 3,038 (88%) white (*n* = 3,460) patients had a history of β-blocker use at discharge (*N* = 4,430). Black patients who received β-blockers survived longer than those not receiving β-blockers and the survival advantage was comparable with white patients (black, adjusted HR = 0.33, 95% CI = 0.23–0.46; white, adjusted HR = 0.48, 95% CI = 0.39–0.58; *p*-for-interaction = 0.74). Among patients discharged on β-blockers, we did not observe a long-term survival advantage for white compared with black patients (HR = 1.2, 95% CI = 0.95–1.5).

**Conclusion:** β-Blocker use at discharge was associated with a survival advantage among black patients after CABG and a similar association was observed in white patients.

## Introduction

Coronary heart disease (CHD) is the cause of 1 in 6 deaths in the United States (1). Over the next 20 years, the prevalence of cardiovascular disease, including CHD, will increase by 10% with a threefold increase in cost (2). Modifiable risk factors (e.g., smoking, diet, obesity) lead to an increased risk of CHD and reductions in CHD mortality have been observed and attributed to public health programs of smoking cessation and the implementation of cholesterol and antihypertensive medications to patients at-risk for the development of CHD (3).

An estimated 397,000 coronary artery bypass grafting (CABG) procedures are performed annually in the United States with an estimated direct and indirect cost approaching $181 billion (4). Between 1980 and 2000, the age-adjusted death rate due to CHD fell by 48% with 50% of this decrease being attributed to secondary preventative measures in patients with known CHD, such as the optimization of medical therapies after myocardial infarction (MI) and revascularization procedures, including CABG (5). Due to the projected increases in CHD prevalence and the known benefit of CABG, the identification of therapies that improve outcomes in the post-surgical population is of paramount importance.

Several studies have reported that black race is associated with decreased long-term survival after CABG compared with whites (6–10). In patients who have undergone CABG, those discharged on β-blockers have been reported to survive longer compared with those not discharged on β-blockers (11–13). However, racial differences in β-blocker efficacy have been reported in heart failure patients, suggesting that these therapies are less effective in black compared with white patients (14). Potentially, similar differences exist in the CABG population who are prescribed β-blockers at the time of discharge. The purpose of this study was to examine the association of discharge β-blockers on long-term survival among black CABG patients and to compare the magnitude of this association with white patients.

## Materials and Methods

This analysis and a waiver of consent were approved by the Institutional Review Board at the Brody School of Medicine, East Carolina University. Details of the study database and methodology have been previously described and are briefly summarized below (15–17).

### Study design

This was a retrospective cohort study of patients undergoing first-time, isolated CABG at the East Carolina Heart Institute between July 2002 and May 2011. Demographic data, comorbid conditions, coronary artery disease (CAD) severity, and surgical data were collected at the time of surgery. Patients who were and were not discharged on β-blockers were compared. Only black and white patients were included to minimize the potential for residual confounding (~1% other races). Racial identity was self-reported. Emergent cases were considered a different population due to clinical instability and were excluded in our analysis (*n* = 98).

### Definitions

A history of β-blocker use at discharge was the exposure variable of interest. Mortality was defined as any cause of death postoperatively. CAD was defined as at least 50% stenosis and confirmed by angiography before surgery.

### Setting

The East Carolina Heart Institute is a population-based tertiary referral heart hospital located in the center of eastern North Carolina, a rural region with a large black population (18). The institute is the largest stand-alone hospital devoted to cardiovascular care in the state of North Carolina, with an emphasis on reducing the unequal burden of cardiovascular disease in this region. The center provides care to patients that predominantly live and remain within a 150-mile radius of the medical center.

### Data collection and follow-up

The primary sources of data were the Society of Thoracic Surgeons Adult Cardiac Surgery Database linked with the electronic medical record at the Brody School of Medicine. The National Death Index was used to obtain death dates for patients lost to follow-up and also used to validate death information captured in our electronic medical record. Linkage with the National Death Index was based on a multiple criteria, deterministic matching algorithm, which included a patient’s social security number (19). In our database, <5% of validated deaths failed to correctly match with the National Death Index. Beginning in 2012, the use of social security numbers as a patient identifier was proscribed within our university system in compliance with §205(*r*) of the Social Security Act (20). Information on β-blocker use at discharge was not collected in our database prior to 2002.

### Statistical analysis

Categorical variables were reported as frequency and percentage while continuous variables were reported as mean ± standard deviation, median, and range. Follow-up time was measured from the date of surgery to the date of death or censoring. Survival probabilities were computed using the Kaplan–Meier product-limit method and stratified by β-blocker status. The log-rank test was used to compare survival between patients with and without discharge β-blockers. Cox proportional hazard regression models were used to compute hazard ratios (HR) and 95% confidence intervals (CI) for long-term mortality. The main model was adjusted for variables that have been previously reported to be associated with cardiovascular-related mortality and included the following covariates: age, sex, race, hypertension, CAD severity, heart failure, and prior stroke. The *post hoc* addition of other variables into the model was performed in a pairwise fashion. *P*-for-interaction between race and discharge β-blocker use was computed using a likelihood ratio test. The test statistic of Grambsch and Therneau was used to check the proportional hazards assumption (21). Temporality during the study period was assessed by stratifying the analysis by two time periods (e.g., 2002–2006, 2007–2011). There were no missing values for the variables used in this analysis. Statistical significance for categorical variables was tested using Fisher’s exact test and the Deuchler–Wilcoxon procedure for continuous variables. Statistical significance was defined as *p* < 0.05. SAS Version 9.3 (Cary, NC, USA) was used for all analyses.

## Results

A total of 853 (88%) black (*n* = 970) and 3,038 (88%) white (*n* = 3,460) patients had a history of β-blocker use at discharge (*N* = 4,430). Patient characteristics are described in Table 1. Preoperative medications and postoperative complications are shown in Tables 2 and 3, respectively. The median follow-up for study participants was 4.3 years.

**Table 1:** **Patient characteristics**.

Characteristic	Black (*n* = 970)		White (*n* = 3,460)	
	β-blocker *n* (%)	No β-blocker *n* (%)	β-blocker *n* (%)	No β-blocker *n* (%)
Overall	853 (88)	117 (12)	3, 038 (88)	422 (12)
Age (years)
Mean ± SD, median (range)	61 ± 10, 60 (33–86)	63 ± 11, 63 (33–86)^†^	64 ± 10, 64 (26–90)	67 ± 10, 68 (28–89)^††^
Male	545 (64)	57 (49)^††^	2, 312 (76)	278 (66)^††^
BMI (kg/m^2^)
Mean ± SD, median (range)	31 ± 6.3, 30 (17–55)	31 ± 7.5, 30 (19–64)	30 ± 5.5, 29 (13–54)	29 ± 6.0, 29 (14–66)
Operative status (elective)	419 (49)	56 (48)	1, 485 (49)	202 (48)
CAD severity
1 Vessel	60 (7)	4 (3)	211 (7)	24 (6)
2 Vessel	208 (24)	35 (30)	797 (26)	102 (24)
3 Vessel	585 (69)	78 (67)	2, 030 (67)	296 (70)
Left main disease	240 (28)	24 (21)	782 (26)	128 (30)
Recent smoker	308 (36)	36 (31)	942 (31)	133 (32)
Hypertension	783 (92)	101 (86)	2, 476 (82)	335 (79)
Diabetes	415 (49)	72 (62)^†^	1, 124 (37)	164 (39)
Heart failure	259 (30)	49 (42)^†^	717 (24)	152 (36)^††^
COPD	148 (17)	28 (24)	618 (20)	134 (32)^††^
Peripheral arterial disease	144 (17)	21 (18)	464 (15)	89 (21)^††^
Dialysis	57 (7)	14 (12)	24 (1)	7 (2)
Prior MI	441 (52)	68 (58)	1, 435 (47)	237 (56)^††^
Prior stroke	110 (13)	19 (16)	222 (7)	31 (7)
Prior PCI	216 (25)	30 (26)	728 (24)	111 (26)

*^†^*p* < 0.05*.

*^††^*p* < 0.01*.

*Statistical significance for categorical variables was tested using Fisher’s exact test and the Deuchler–Wilcoxon procedure for continuous variables*.

*BMI, body mass index; CAD, coronary artery disease; COPD, chronic obstructive pulmonary disease; MI, myocardial infarction; PCI, percutaneous coronary intervention; SD, standard deviation*.

**Table 2:** **Preoperative medications**.

Medication	Black (*n* = 970)		White (*n* = 3,460)	
	β-blocker *n* (%)	No β-blocker *n* (%)	β-blocker *n* (%)	No β-blocker *n* (%)
Preoperative β-blocker	691 (81)	81 (69)^††^	2, 358 (78)	276 (65)^††^
Aspirin	665 (78)	83 (71)	2, 472 (81)	318 (75)^††^
Lipid-lowering agents	634 (74)	79 (68)	2, 236 (74)	275 (65)^††^
Anticoagulants	309 (36)	48 (41)	1, 120 (37)	163 (39)
Antiplatelet agents	175 (21)	36 (31)^†^	686 (23)	121 (29)^††^
Calcium channel blockers	213 (25)	36 (31)	536 (18)	90 (21)
Diuretics	230 (27)	36 (31)	582 (19)	126 (30)^††^
ACE inhibitors/ARBs	469 (55)	54 (46)	1, 309 (43)	189 (45)
Digitalis	23 (3)	13 (11)^††^	96 (3)	27 (6)^††^
Nitrates	89 (10)	19 (16)	367 (12)	54 (13)
Inotropic agents	7 (1)	4 (3)^†^	24 (1)	10 (2)^††^

*^†^*p* < 0.05*.

*^††^*p* < 0.01*.

*Statistical significance for categorical variables was tested using Fisher’s exact test and the Deuchler–Wilcoxon procedure for continuous variables*.

*ACE, angiotensin converting enzyme; ARB, angiotensin receptor blocker*.

**Table 3:** **Postoperative complications**.

Complication	Black (*n* = 970)		White (*n* = 3,460)	
	β-blocker *n* (%)	No β-blocker *n* (%)	β-blocker *n* (%)	No β-blocker *n* (%)
MI	0 (0)	1 (1)	6 (< 1)	3 (1)
Stroke	15 (2)	8 (7)^††^	18 (1)	25 (6)^††^
ARDS	4 (<1)	5 (4)^††^	12 (< 1)	31 (7)^††^
Pneumonia	16 (2)	8 (7)^††^	35 (1)	37 (9)^††^
GI event*	40 (5)	11 (9)^†^	58 (2)	40 (9)^††^
Renal failure	18 (2)	10 (9)^††^	38 (1)	39 (9)^††^

*^†^*p* < 0.05*.

*^††^*p* < 0.01*.

*Statistical significance for categorical variables was tested using Fisher’s exact test and the Deuchler–Wilcoxon procedure for continuous variables*.

*Includes GI bleed, pancreatitis, cholecystitis, mesenteric ischemia, and other GI events.

*ARDS, acute respiratory distress syndrome; GI, gastrointestinal; MI, myocardial infarction*.

**Figure 1:**
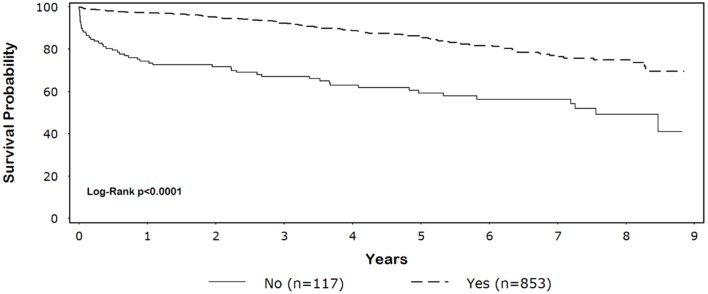
**Unadjusted Kaplan–Meier survival among black patients by β-blocker discharge status**.

**Figure 2:**
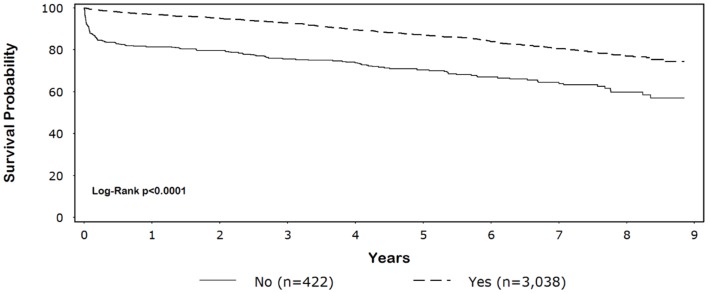
**Unadjusted Kaplan–Meier survival among white patients by β-blocker discharge status**.

Five-year survival for black patients with and without discharge β-blockers was 86% and 59%, respectively (*p* < 0.0001) (Figure 1). In whites, 5-year survival for patients discharged on β-blockers was 87% compared with 70% for patients without discharge β-blockers (*p* < 0.0001) (Figure 2).

CABG patients receiving β-blockers at discharge survived significantly longer than those not receiving β-blockers (blacks: unadjusted HR = 0.31, 95% CI = 0.22–0.43, adjusted HR = 0.33, 95% CI = 0.23–0.46; whites: unadjusted HR = 0.40, 95% CI = 0.33–0.48; adjusted HR = 0.48, 95% CI = 0.39–0.58; *p*-for-interaction = 0.74). Among patients discharged on β-blockers, we did not observe a long-term survival advantage for white compared with black patients (HR = 1.2, 95% CI = 0.95–1.5). The main result was not substantively altered (±10% change in effect size) with the pairwise addition of other variables listed in Table 1 or preoperative β-blockers.

## Discussion

A survival benefit was observed among black CABG patients who received discharge β-blockers and the magnitude of this association was comparable with white patients. Recent studies have focused on the impact of preoperative β-blockers and their influence on short-term mortality (22, 23). Only a few studies have looked at the impact of these medications at discharge on long-term survival after CABG (11–13).

An investigation of 3,102 patients discharged on β-blockers after cardiac surgery reported that patients using these medications were more likely to be alive 6 years after surgery compared with those not discharged on β-blockers (adjusted HR = 0.65, 95% CI = 0.49–0.87) (12). While the majority of patients included in this study were isolated CABG, HRs were not provided by type of surgery. Additionally, racial differences were not examined in this study, possibly due to the limited diversity of surgical populations in western Canada.

β-Blockers were found to improve 1-year outcomes in patients with chronic obstructive pulmonary disease (adjusted HR = 0.38, *p* = 0.003) (13). In contrast with the current study, racial differences were not reported and study populations were limited to patients with chronic obstructive pulmonary disease. Additionally, patients undergoing CABG in the PREVENT IV trial were surveyed for use of secondary prevention medications (e.g., antiplatelet agents, β-blockers, angiogtensin-converting enzyme inhibitors or angiotensin receptor blockers, and lipid-lowering agents) 1 year after hospital discharge and cardiac-related mortality was assessed at 2 years (11). Although not statistically significant, patients discharged with β-blockers observed a trend of improved survival than those not receiving β-blockers (adjusted HR = 0.53, 95% CI = 0.24–1.17). A survival advantage was not observed in post-CABG patients who were not ideal candidates for β-blockers (adjusted HR = 1.19, 95% CI = 0.70–2.01).

Racial differences in the effectiveness of β-blockers in cardiovascular patients have been reported in the literature. A 2-year follow-up study of heart failure patients receiving β-blockers observed that white patients benefited more than black patients (black: adjusted HR = 0.67, 95% CI = 0.48–0.94; white: adjusted HR = 0.40, 95% CI = 0.27–0.60) (14). However, we did not observe a statistically significant survival advantage for white patients receiving β-blockers compared with blacks. Known genetic polymorphisms in the β-adrenergic pathway possibly explain reported inconsistencies in the literature (24, 25).

### Strengths and limitations

Our study is strengthened by its large racially dichotomous sample size and long-term follow-up. Furthermore, we were able to accurately determine time of death using a combination of the National Death Index and our comprehensive electronic medical record.

Data regarding socioeconomic position, education, and income were not collected and these factors potentially influenced survival (26). Alcohol consumption also was not recorded in our dataset, but this variable has not been associated with increased morbidity and mortality after CABG (27). We did not have information on long-term β-blocker use. Adherence to medication among study participants may have changed. In general, β-blocker adherence has been shown to be relatively stable after CABG and any non-adherence to β-blockers after discharge likely would have biased results toward the null, decreasing the probability of finding an association of β-blocker use and long-term survival (28). However, a statistically significant result was observed in our study despite potential non-adherence to β-blocker therapies.

Physicians may have prescribed β-blockers to patients perceived to have better prognoses, potentially introducing selection bias into the analysis. However, the association of discharge β-blocker use and lower mortality was preserved after excluding patients who died within the first 30 days of surgery (black: adjusted HR = 0.42, 95% CI = 0.28–0.61; white: adjusted HR = 0.71, 95% CI = 0.56–0.90). Presumably, the accuracy of selecting patients who will survive diminishes following the postoperative period. While β-blocker therapy is relatively contraindicated in patients with chronic obstructive pulmonary disease and may have resulted in selection bias, only a small number of these patients were not discharged on β-blockers and this percentage was similar between races. β-Blocker use also is relatively contraindicated in heart failure patients with depressed ejection fraction (< 0.35). However, few patients with depressed ejection fraction who did not receive β-blockers were in our dataset (black, 2.5%; white, 2.2%). Excluding these patients did not substantively alter our findings (black, adjusted HR = 0.29, 95% CI = 0.20–0.43; white, adjusted HR = 0.48, 95% CI = 0.38–0.60).

Patients in this study were recruited over a relatively long period (~10 years), during which practice methods and clinical care may have changed. However, adjusting for study period had little impact on our findings. Similarly, the status of several variables in our analysis may have changed over time (e.g., medications and postoperative complications). We did not adjust for these variables in a time-dependent manner due to their potential to be in the causal pathway. Cause of death is not recorded in the National Death Index and β-blocker use may have been unrelated to their mortality. Although we adjusted for known clinically relevant variables, we acknowledge that other unmeasured factors may have resulted in residual confounding. Additionally, the data reported are from a single center and may reflect a chance finding specific to our institution and region.

Multivariable Cox regression models were used to adjust for confounding because of potential “non-collapsibility bias” inherent to propensity scores based on log-linear models and the possible decrease in power due to incomplete matching (29). Additionally, a random forest algorithm (e.g., machine learning) was not used for matching. This algorithm potentially introduces misspecification bias into the propensity score model due to the “black box” nature of the algorithm that obscures the etiologic relationship between predictors and outcome (30, 31).

In conclusion, β-blocker use at discharge was associated with a survival advantage among black patients after CABG and the magnitude of the effect was similar to that observed among white patients. In contrast with prior studies that have reported β-blocker use is less effective in black patients, we did not observe a similar association at our institution among black CABG patients discharged on β-blockers. Additional studies are needed to better understand the underlying mechanisms of our results and to appropriately target future treatment strategies among CABG patients.

## Conflict of Interest Statement

The authors declare that the research was conducted in the absence of any commercial or financial relationships that could be construed as a potential conflict of interest.
